# Development of SkinTracker, an integrated dermatology mobile app and web portal enabling remote clinical research studies

**DOI:** 10.3389/fdgth.2023.1228503

**Published:** 2023-09-06

**Authors:** Joy Q. Jin, Julie Hong, Kareem G. Elhage, Mitchell Braun, Riley K. Spencer, Mimi Chung, Samuel Yeroushalmi, Edward Hadeler, Megan Mosca, Erin Bartholomew, Marwa Hakimi, Mitchell S. Davis, Quinn Thibodeaux, David Wu, Abhilash Kahlon, Paul Dhaliwal, Erin F. Mathes, Navdeep Dhaliwal, Tina Bhutani, Wilson Liao

**Affiliations:** ^1^ School of Medicine, University of California, San Francisco, San Francisco, CA, United States; ^2^ Department of Dermatology, University of California, San Francisco, San Francisco, CA, United States; ^3^ RedBlink Inc., Danville, CA, United States

**Keywords:** atopic dermatitis, biometric data acquisition, clinical research study, eczema, inflammatory skin disease, mobile application, remote clinical research, smartwatch

## Abstract

**Introduction:**

In-person dermatology clinical research studies often face recruitment and participation challenges due to travel-, time-, and cost-associated barriers. Studies incorporating virtual/asynchronous formats can potentially enhance research subject participation and satisfaction, but few mobile health tools are available to enable remote study conduct. We developed SkinTracker, a patient-facing mobile app and researcher-facing web platform, that enables longitudinal collection of skin photos, patient reported outcomes, and biometric health and environmental data.

**Methods:**

Eight design thinking sessions including dermatologists, clinical research staff, software engineers, and graphic designers were held to create the components of SkinTracker. Following iterative prototyping, SkinTracker was piloted across six adult and four pediatric subjects with atopic dermatitis (AD) of varying severity levels to test and provide feedback on SkinTracker for six months.

**Results:**

The SkinTracker app enables collection of informed consent for study participation, baseline medical history, standardized skin photographs, patient-reported outcomes (e.g., Patient Oriented Eczema Measure (POEM), Pruritus Numerical Rating Scale (NRS), Dermatology Life Quality Index (DLQI)), medication use, adverse events, voice diary to document qualitative experiences, chat function for communication with research team, environmental and biometric data such as exercise and sleep metrics through integration with an Apple Watch. The researcher web portal allows for management and visualization of subject enrollment, skin photographs for examination and severity scoring, survey completion, and other patient modules. The pilot study requested that subjects complete surveys and photographs on a weekly to monthly basis via the SkinTracker app. Afterwards, participants rated their experience in a 7-item user experience survey covering app function, design, and desire for participation in future studies using SkinTracker. Almost all subjects agreed or strongly agreed that SkinTracker enabled more convenient participation in skin research studies compared to an in-person format.

**Discussion:**

To our knowledge, SkinTracker is one of the first integrated app- and web-based platforms allowing collection and management of data commonly obtained in clinical research studies. SkinTracker enables detailed, frequent capture of data that may better reflect the fluctuating course of conditions such as AD, and can be modularly customized for different skin conditions to improve dermatologic research participation and patient access.

## Introduction

Clinical research in dermatology often involves visual assessment of the skin. As a prototype skin disease, atopic dermatitis (AD) is the most common chronic inflammatory skin condition with a lifetime prevalence of nearly 20%, affecting both children and adults (1). Between 2019 and 2021, over 20 randomized clinical trials (RCTs) were conducted investigating systemic immunomodulatory medications for AD treatment; each required in-person visual skin assessment of research subjects to determine the degree of AD severity and treatment-associated improvements (2). These RCTs serve as the gold standard for understanding how new therapies improve patient outcomes and quality of life (QoL).

However, RCTs and other types of in-person studies (e.g., case-control, cohort, registry studies) can be hampered by several difficulties. Challenges with patient recruitment represent the single biggest cause of clinical trial delays, which occur in over 80% of RCTs, with 94% delayed by at least one month (3, 4). Delays of just one month have been associated with potential losses of $600,000 per day, depending on the extent of wasted time, resources, and unusable patient data (5, 6). In-person studies are also challenging for participants due to logistic, financial, and travel reasons. Therefore, alternative or supplementary ways to improve patient participation and retention are urgently needed.

Hybrid and remote (i.e., virtual, decentralized) clinical trials have emerged as a promising strategy to tackle this problem. The financial savings associated with virtual studies are achieved through shorter enrollment periods, faster data collection, ability to manage multiple study sites virtually, and reduced commuting/lost productivity for patients (3). These factors can significantly reduce costs associated with in-person clinical studies, which total 6 billion USD annually for patient recruitment and an average of 2.6 billion USD per clinical trial (3). For example, one European study found remote clinical visits to be associated with savings of over 650 USD per AD patient within one year of treatment alone, primarily lowering costs via reduced work absenteeism (7). Virtual visits can enable patients who otherwise face difficulties attending in-person visits to access clinical studies, and increase the ethnoracial and geographic diversity of participants compared to purely clinic-based studies, improving the generalizability of results that inform evidence-based medical care (8). Despite the potential advantages of incorporating virtual components into dermatology clinical research, few published AD clinical trials have incorporated mobile digital data collection (e.g., via smartphone or smartwatch) into the study design (9).

One of the biggest barriers to conducting remote dermatologic research is the lack of tools designed to collect patient data in a format that meets clinical research standards (10). With AD, a handful of digital applications (i.e., apps) have been published to help educate patients and manage their disease (10–14), but only one mobile app was specifically designed for clinical research purposes (14) and primarily collected itch severity ratings, coupled with a non-validated quality-of-life survey. In the clinical realm, the need for more comprehensive remote clinical monitoring tools for AD and other inflammatory skin diseases is particularly high, as patients often experience skin fluctuations or flares between in-person visits, meaning such encounters may not fully capture patients' clinical journeys while attempting new treatments (1). The development of a mobile app empowering clinical research participation and asynchronous review of subject outcomes would contribute greatly towards the study of AD. Ideally, this digital solution could be built in a modular way expandable to other skin conditions with fluctuating disease courses, such as psoriasis (15).

Here, we report the development of SkinTracker, a patient-facing mobile app with associated researcher-facing web portal, designed with the input of dermatologists, clinical research staff, software engineers, graphic designers, and AD patients. We describe the design and functions of the app, which include capture of patient-reported outcome measures (PROMs) via validated survey tools frequently used in AD clinical studies, patient-submitted photographs to track AD skin severity, and smartwatch-synced biometric and environmental data, all reviewable via a researcher web portal. SkinTracker function and usability were further refined based on pilot testing with ten adult and pediatric AD patients.

## Materials and methods

This single-center, investigator-initiated observational study was approved by the University of California San Francisco (UCSF) Institutional Review Board (IRB #19-28676). Informed consent was obtained from the participants and their parent/guardian in the event of a minor's participation.

SkinTracker app development was conducted by two dermatologists (UCSF, San Francisco, CA), nine clinical research staff (UCSF, San Francisco, CA), and three software engineers and graphic designers (Redblink, Inc., Dublin, CA). Subjects piloting the app included ten participants aged 13 years and up diagnosed by a dermatologist with AD.

### SkinTracker app and physician portal development

The SkinTracker integrated system was developed over 16 months (November 2020 through March 2022) prior to subject recruitment for pilot testing. During initial meetings, the mobile app goals were defined based on three domains: (1) Overarching goals—to enable remote research studies of the skin and increase accessibility of research for patients not near a research institution, (2) App functionality for patients—provide clear instructions in a checklist format for study completion, enable longitudinal input of AD survey responses and skin photographs, and enable communication with study providers, and (3) Web portal functionality for researchers—manage screening, consent, and enrollment, monitor patients’ medication changes and study task progress, communicate with patients, and visualize data for analysis.

Eight design thinking sessions hosted by the dermatologists, clinical research staff, and software developers were then held between December 2020 and August 2021 to outline the possible modules for inclusion in the app. The goals of these meetings were to develop the structure and interface of the SkinTracker app for study subjects and researchers, align with institutional security/compliance guidelines for human subjects' research, ethics committee, and informational technology, outline data storage plans according to HIPAA guidelines, and incorporate PROMs. This process included a literature review of telehealth studies incorporating both patient- and provider-submitted store-and-forward images for adults and pediatric patients (16–24), and clinical research studies incorporating biometric fitness data from smartwatches and wearable monitors (25–37). Following the design thinking sessions, the software development team underwent a design sprint process to prototype and test the proposed ideas for inclusion in the app prototype (i.e., wireframe) (38). Input from physicians and other stakeholders were obtained for both the researcher- and study subject-facing components of SkinTracker. The information collected was synthesized to inform the development of the modules included in the integrated app and researcher portal used for pilot testing.

### Data security and privacy

SkinTracker patient users were required to complete consent (if 18 years and up) or assent (13–17 years old; also signed by parent/legal guardian) forms approved by UCSF (IRB #19-28676) detailing study participation, overview, and rights. Under HIPAA requirements, the Permission to Use Personal Health Information for Research provided by the University of California and Bill of Rights for Experimental Subjects were two additional forms signed by all users to enable collection of skin photographs, quality-of-life, and smartwatch monitoring data. In conjunction with the Information Technology (IT) department at UCSF, a data diagram was constructed to outline the flow of collected patient data through SkinTracker (Supplementary Figure 1). Furthermore, an IT questionnaire was completed to identify the level of researcher and vendor access to SkinTracker, data classification types, regulations adhered to, and Control of Access to and Release Information form. All data collected through SkinTracker mobile app and analyzed through the researcher web portal was securely hosted on UCSF servers. Encrypted password login was required for both mobile app users and researchers, with identifying information (e.g., email, password, username) stored on an encrypted database. Patient information saved for further analyses (e.g., photographs, graphs) were de-identified and stored on a secure share drive hosted by UCSF, only accessible by research team members granted access.

### Participant recruitment and pilot testing

Ten subjects with AD were recruited for a six-month, hybrid in-person and remote pilot test of the SkinTracker integrated system. Subjects included: three subjects over 18 years with an Eczema Area and Severity Index (EASI) score ≥10 and Investigator Global Assessment (IGA) ≥3; three subjects over 18 years with an EASI score between 1 and 10 and IGA of 1 to 2; two subjects between 13 and 17 years with an EASI score ≥10 and IGA ≥3; two subjects between 13 and 17 years with an EASI score between 1 and 10 and IGA of 1 to 2. Subjects must have been at least 13 years of age with the ability to provide written informed consent and comply with the protocol, with a formal diagnosis of AD by a dermatologist for at least six months, and owned or had access to an iPhone device compatible with the SkinTracker mobile app. Exclusion criteria included those with a history of immunosuppression, history of malignancy within five years of the screening visit (except cervical carcinoma *in situ* or non-metastatic squamous or basal cell carcinoma of the skin if all were completely treated and resolved), severe concomitant illness, or serious known infection.

Subjects were recruited between April and November 2022 via in-person clinical visits and retrospective chart review of pediatric and adult dermatology clinic patients at UCSF, and physical recruitment flyers posted within UCSF clinical spaces. Subjects were not administered any drug or medication as part of this study; their AD treatments were prescribed by healthcare providers outside of the study per standard of care and these providers maintained discretion over changes in AD treatment. Subjects were financially compensated for their participation.

Data collection during pilot testing occurred from May 2022 through May 2023. Pilot subjects were educated by clinical research staff at the first in-person visit (Month 0), during which subjects completed the SkinTracker mobile screening and intake surveys and were provided the necessary photography equipment (phone tripod stand, Bluetooth-enabled remote, blue background tarp, supporting tripod frame with clips) and Apple Watch 7 used to track biometric data. Apple Watch setup and pairing with the patient's iPhone and SkinTracker mobile app was completed with the assistance of clinical research staff.

At the conclusion of pilot testing, patients completed a 7-item user experience survey that included components from the mHealth Apps Usability Questionnaire (MAUQ) (user version for interactive apps), a 20-item survey that assesses usability and usefulness of a mobile health app, the user-version of the Mobile App Rating Scale (MARS), and questions specific to the SkinTracker interface (39, 40). The MAUQ and MARS are measurement tools found to be two of the most suitable validated instruments assessing mobile health app quality in a recent systematic review (41).

### Data analysis

Demographic, clinical, and Apple Watch data from pilot participants were securely stored at UCSF. With SkinTracker data collected, photograph EASI and IGA scores were directly compared with those obtained during in-person visits. Individual questions present in quality-of-life surveys (POEM, DLQI) were automatically tallied and weighted based on the respective scoring algorithms using the SkinTracker researcher web portal to generate presented scores. Smartwatch exercise activity (steps, exercise, move, stand) were generated daily, for which the mean was obtained and graphed. A secure Qualtrics survey was administered to all pilot subjects described above, for which the mean and standard deviation (SD) were calculated for each question.

Finally, image quality rating of patient-submitted photographs was conducted using the framework developed by ImageQX (42). ImageQX is a convolutional neural network for image quality assessment specifically developed for teledermatology and includes input from 12 dermatologists and a dataset of 36,509 skin photographs obtained from 2017 to 2019 using a mobile skin disease tracking app incorporating patients internationally (42). While ImageQX is not widely available to the scientific community yet, the principles upon which it was developed was used for evaluation of patient-submitted store-and-forward dermatologic photos in our study, as such evaluation is not currently standardized within the dermatologic field. SkinTracker photographs were rated across the five domains described in ImageQX: bad framing (image not centered on the skin lesion), bad lighting (image was too bright or dark), blur (image with motion blur or inadequate focus), distance issue (image taken from afar with no discernable details), and low resolution (image taken with a low-resolution camera) (42). Three authors (JQJ, KGE, MSD) independently rated all patient-submitted photographs along these five axes, followed by verification (by WL) of any discrepancies.

## Results

### Features of SkinTracker iOS app

**Figure 1:**
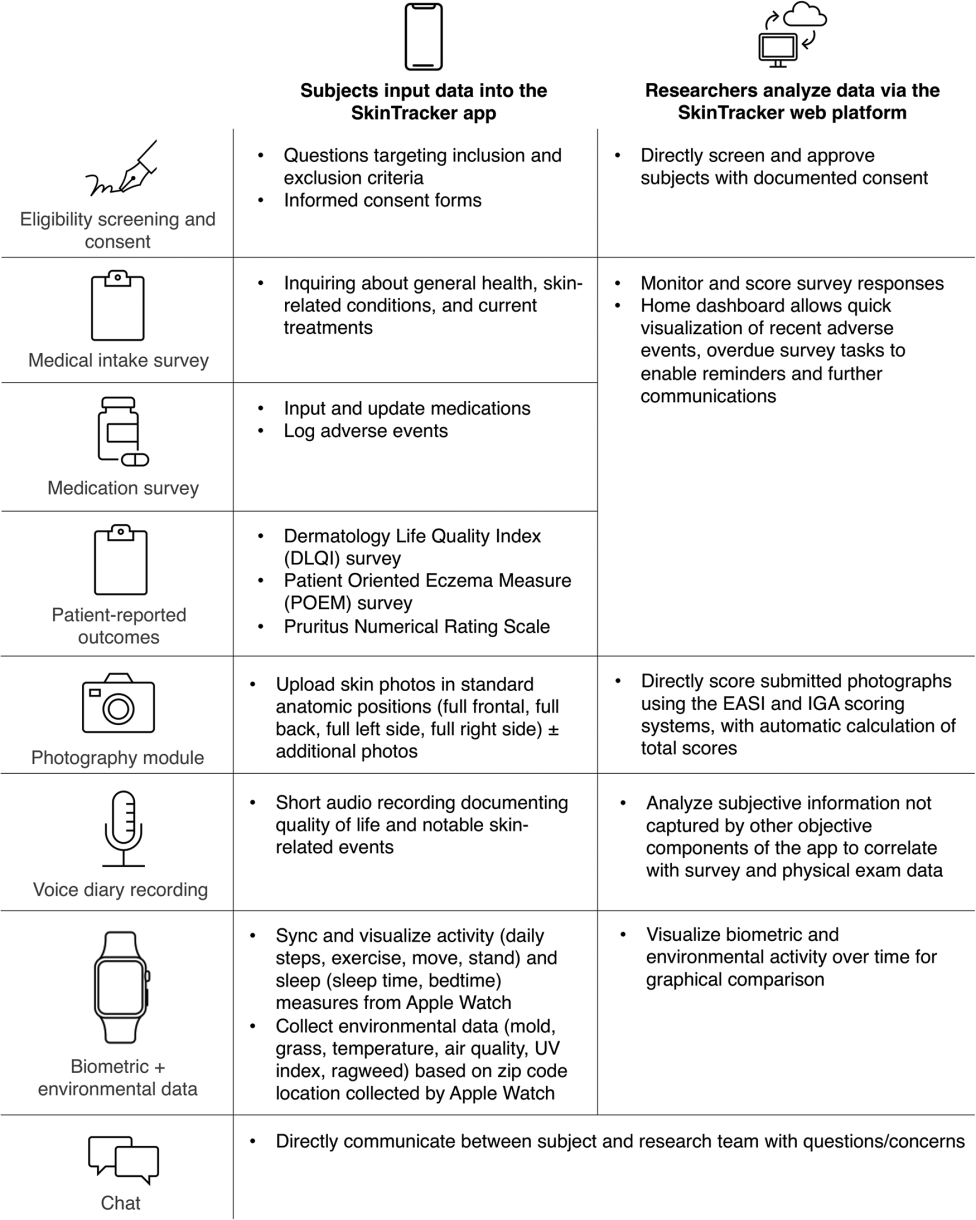
Graphical overview of the SkinTracker integrated system. Summarizes the functionality for research subjects (SkinTracker app) and researchers (SkinTracker web platform). DLQI, Dermatology Life Quality Index; EASI, Eczema Area and Severity Index; IGA, Investigator's Global Assessment; POEM, Patient Oriented Eczema Measure.

**Figure 2:**
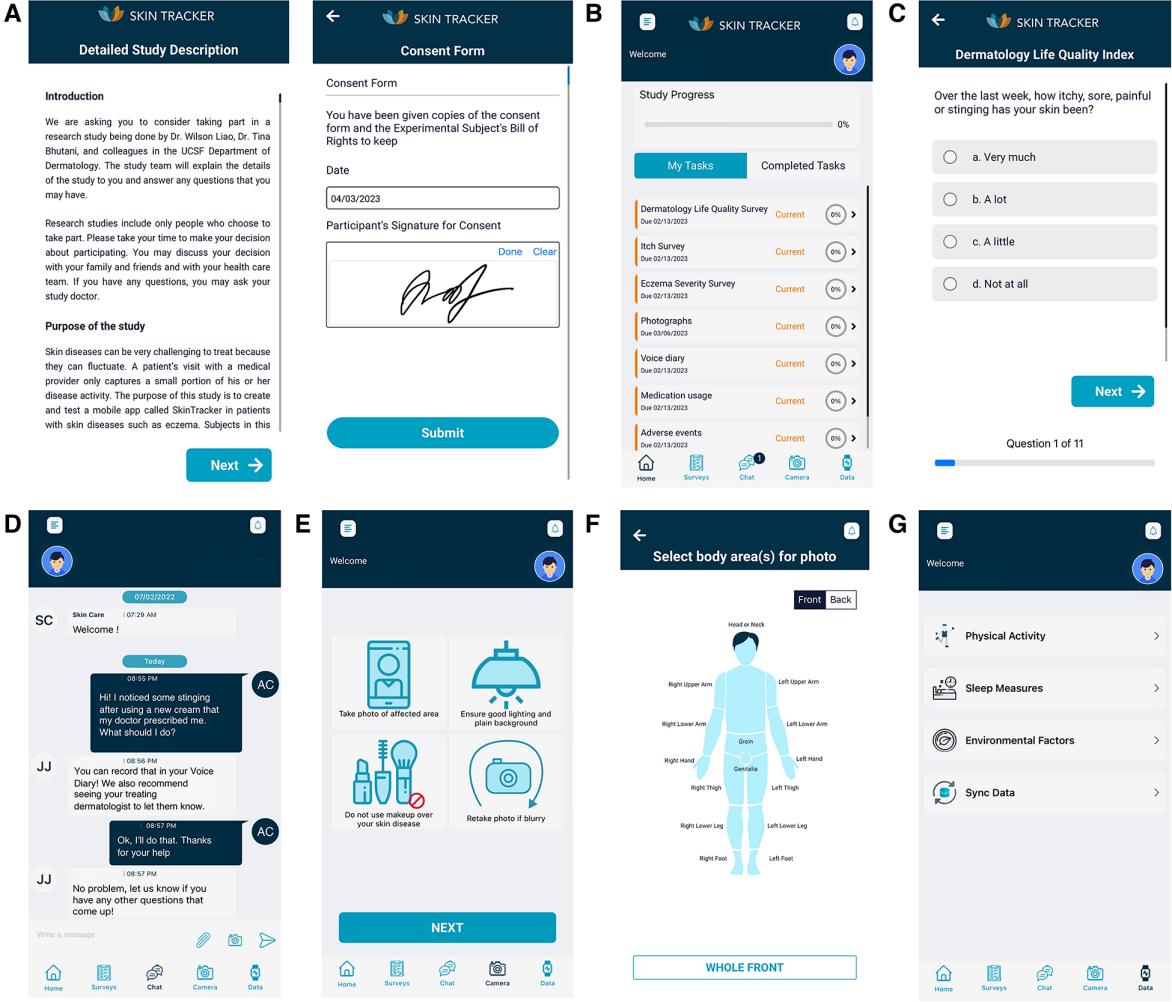
SkinTracker research subject user interface. (**A**) Sample screenshots of the consent form and signature input pages of research subjects enrolling in an eligible study on the SkinTracker app—in this case, the SkinTracker pilot study. (**B**) Landing page of the SkinTracker mobile app, once patients have passed the screening questionnaire and completed all required consent forms. (**C**) Sample survey question displayed from the Dermatology Life Quality Index (DLQI) weekly research task. (**D**) Chat function integrated into the mobile app allowing for communication with clinical research staff on the study team. (**E,F**) App users are instructed on how to use their provided camera backdrop and take monthly photographs of their skin in standard anatomic positions. (**G**) App users can sync data from their Apple smartwatch to longitudinally monitor physical activity, sleep, and environmental metrics within the app, which provides graphic visualization of this data.

The SkinTracker app was designed to include research subject and researcher functions required across the breadth of dermatology clinical research studies (Figure 1). After secure two-factor login, subjects are presented with a study description and informed consent forms, which can be signed digitally (Figure 2A). Next, users answer a screening survey to determine their eligibility for the study. Once approved by the research team for enrollment, users enter data on their medical history and current medication usage. They are then taken to the app's landing homepage, which displays the user's study progress, upcoming tasks with associated due dates, and completed tasks (Figure 2B). Tasks for user completion include surveys inquiring about medication review and adverse events, as well as standardized instruments in AD research such as the Patient Oriented Eczema Measure (POEM) measuring AD severity (43), pruritus Numerical Rating Scale (NRS) (44, 45), and Dermatology Life Quality Survey (DLQI) (46) (survey questions listed in Supplementary Methods; sample screenshot of DLQI question shown in Figure 2C). Users are reminded of study tasks via push notifications automatically sent through the mobile app prior to deadlines. A secure chat function (Figure 2D) allows communication between the user and research team. Additionally, a voice diary function allows patients to submit audio recordings at any timepoint describing their skin (e.g., “the colder temperature over the last few days seemed to worsen my eczema, making my skin feel itchier”).

**Figure 3:**
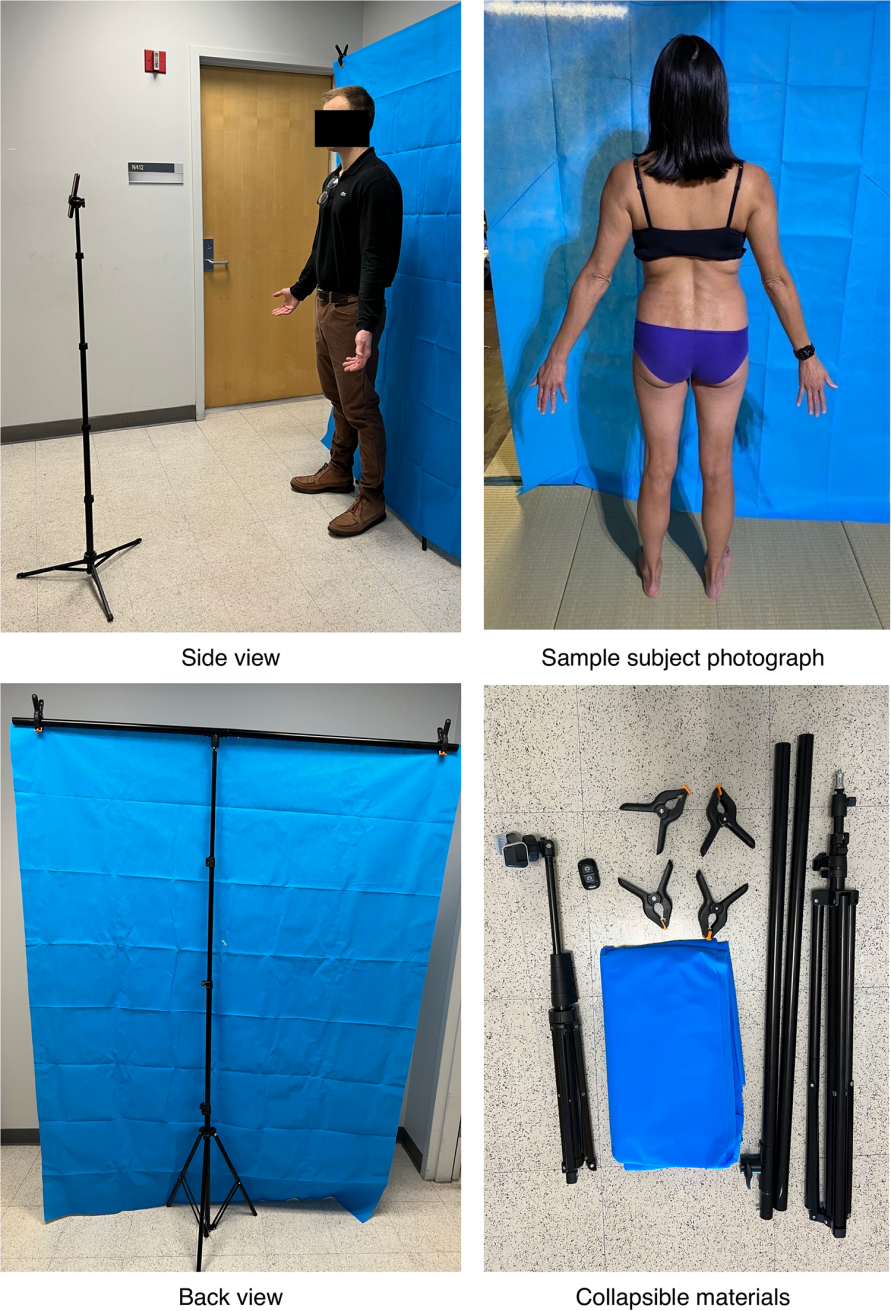
SkinTracker research subject photography capture setup. Demonstrates the positioning of all photography-related equipment that subjects were provided as part of the SkinTracker pilot study, enabling submission of store-and-forward photographs for researcher evaluation and scoring. The collapsible equipment included a smartphone tripod stand, Bluetooth-enabled remote, blue background tarp, and supporting frame with clips.

Users can upload photographs of full-body skin images in standard anatomic positions (full frontal, full back, full left side, full right side) via the app. Prior to initial photo capture and submission, users are presented with an educational video on photography setup and step-by-step text with graphic instructions regarding body positioning and room lighting requirements to facilitate standardized image capture across patients (Figure 2E). In addition to standard anatomic positions, optional photographs of any body part can be uploaded by the user at any time to capture evolving skin lesions or close-up images of specific areas (Figure 2F). Photographs are taken using a phone tripod stand, Bluetooth-enabled remote, blue background tarp, and supporting tripod frame with clips provided as part of the pilot study, enabling subject completion of the research task without assistance from other individuals (Figure 3). The photos can be asynchronously evaluated by the research team for EASI and IGA scoring, which are common skin disease severity outcomes collected in AD research studies (47, 48).

Finally, SkinTracker allows for the user to sync biometric data from their Apple Watch to the app (Figure 2G); this information is viewable by both users and researchers in the SkinTracker app and web portal, respectively. The data captured includes number of daily steps, duration of exercise in minutes, move (number of times the user stood and moved, as tracked by the Apple Watch), number of hours standing, and sleep measures (number of hours slept per night). In addition, environmental data (e.g., temperature, air quality, ultraviolet index) can be collected based on the zip code associated with synced Apple Watch activity.

### Features of SkinTracker researcher-facing web portal

**Figure 4:**
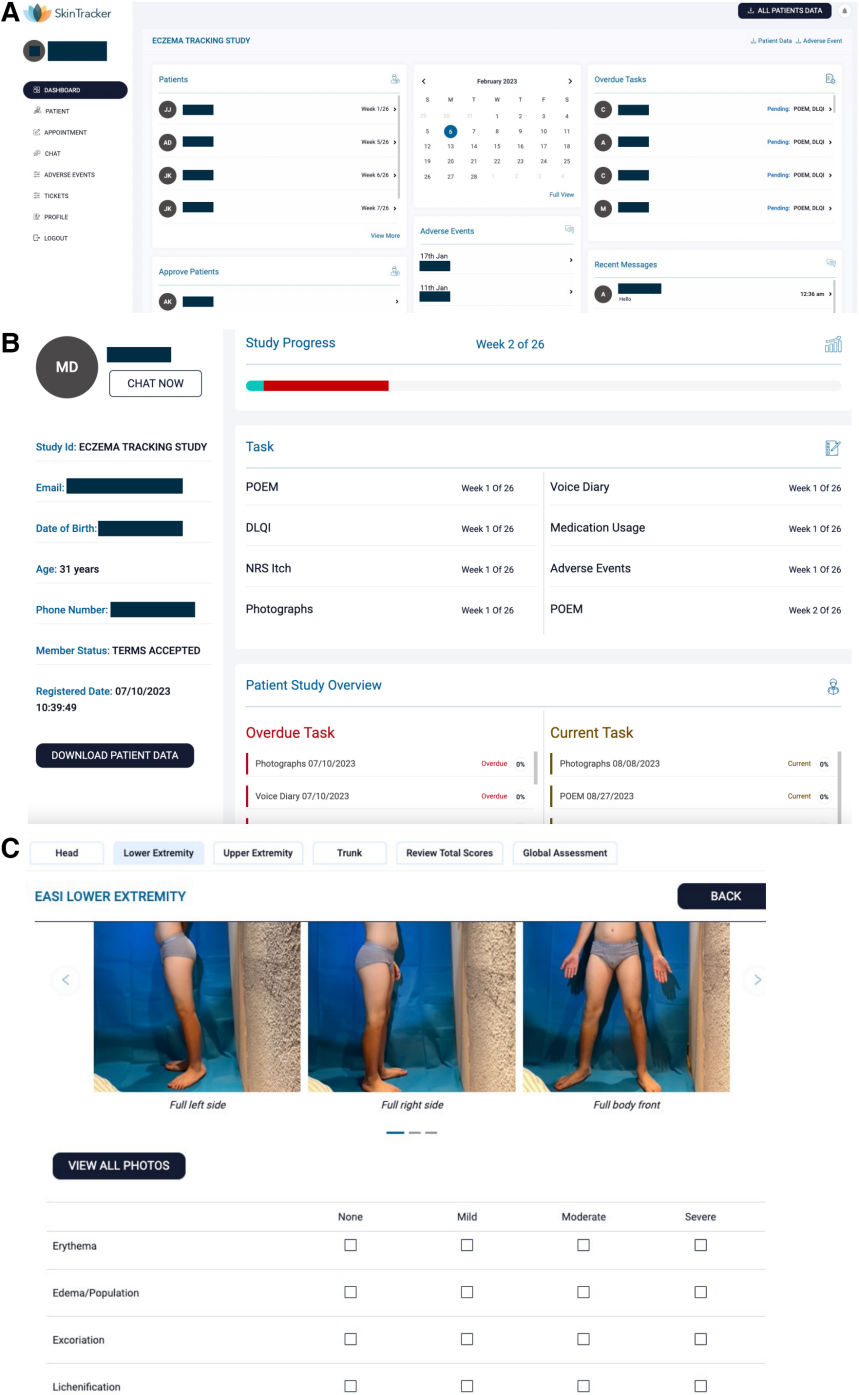
SkinTracker researcher portal user interface. (**A**) Following login to their secure account, physicians are taken to the dashboard (homepage) of the SkinTracker web portal for a specific clinical study of interest (in this case, the pilot study). The dashboard includes an overview of participating subjects, pending subjects who require researcher approval for participation, adverse events and overdue tasks that may require physician follow-up, a study calendar, and recent messages communicated to the physician from subjects using the SkinTracker app's chat function. The dashboard also allows for bulk download of subject data for study analysis. (**B**) Sample overview of a subject dashboard that physicians can view. The dashboard includes the ability to view completed, overdue, and upcoming research tasks sent to the subject's SkinTracker mobile app. Physicians can also view responses to the voice diary, survey or photography tasks, activity data, and chat conversations via navigation of the bottom pane. (**C**) Sample view of patient-submitted photographs during Month 1 of the pilot study, where physicians can expand, zoom in/out, and download specific images, and score the subject's skin exam through the Eczema Area and Severity Index (EASI) and Investigator Global Assessment (IGA).

An integrated researcher-facing web portal was developed in conjunction with the SkinTracker app to ensure that researchers could monitor data from and interact with subjects. Following a secure web login, the researcher is taken to the SkinTracker portal dashboard, which provides a summary overview of all enrolled subjects, overdue research tasks (to enable researchers to send mobile app reminders to subjects), adverse events, and recent messages sent through the secure chat function (Figure 4A). Researchers can then select individual subjects to view study progress, including completed, upcoming, and overdue survey and photography tasks (Figure 4B). Selection of individual tasks allows a detailed view of the subject's responses; bulk download of survey, smartwatch, and photography data is also possible for individual subjects and across all study participants. Smartwatch data includes graphs visualizing subjects' physical activity and sleep measures over time. Additionally, a function allowing researchers to analyze user-submitted photographs for calculation of EASI and IGA scores per photo set was created (Figure 4C). These functionalities enable remote researcher analyses of objective measures such as smartwatch-reported activity steps and duration of sleep with self-reported itch and quality-of-life via validated surveys; this information can be correlated with objective physician-scored EASI and IGA by timepoint.

### Pilot testing and user experience

Following the development of the integrated SkinTracker system, a six-month pilot testing phase in ten AD subjects was conducted to evaluate usability and incorporate feedback. One additional adult subject was initially enrolled but withdrew from the study following five weeks of participation due to personal reasons. The mean age of the six adult subjects was 29.6 [standard deviation (SD), 8.1] years, while the mean age of the four pediatric subjects was 15.3 (SD, 2.1) years. Subjects included six male, four female, one White, eight Asian, and one Hispanic individual; baseline BSA included subjects with <5% (three subjects), 5–10% (two subjects), 10–20% (two subjects), and >20% (three subjects). No subjects reported cigarette use and mean BMI was 24.5 (SD, 4.2). Alcohol use varied (three reported no use, six reported occasional use, one reported frequent use).

**Figure 5:**
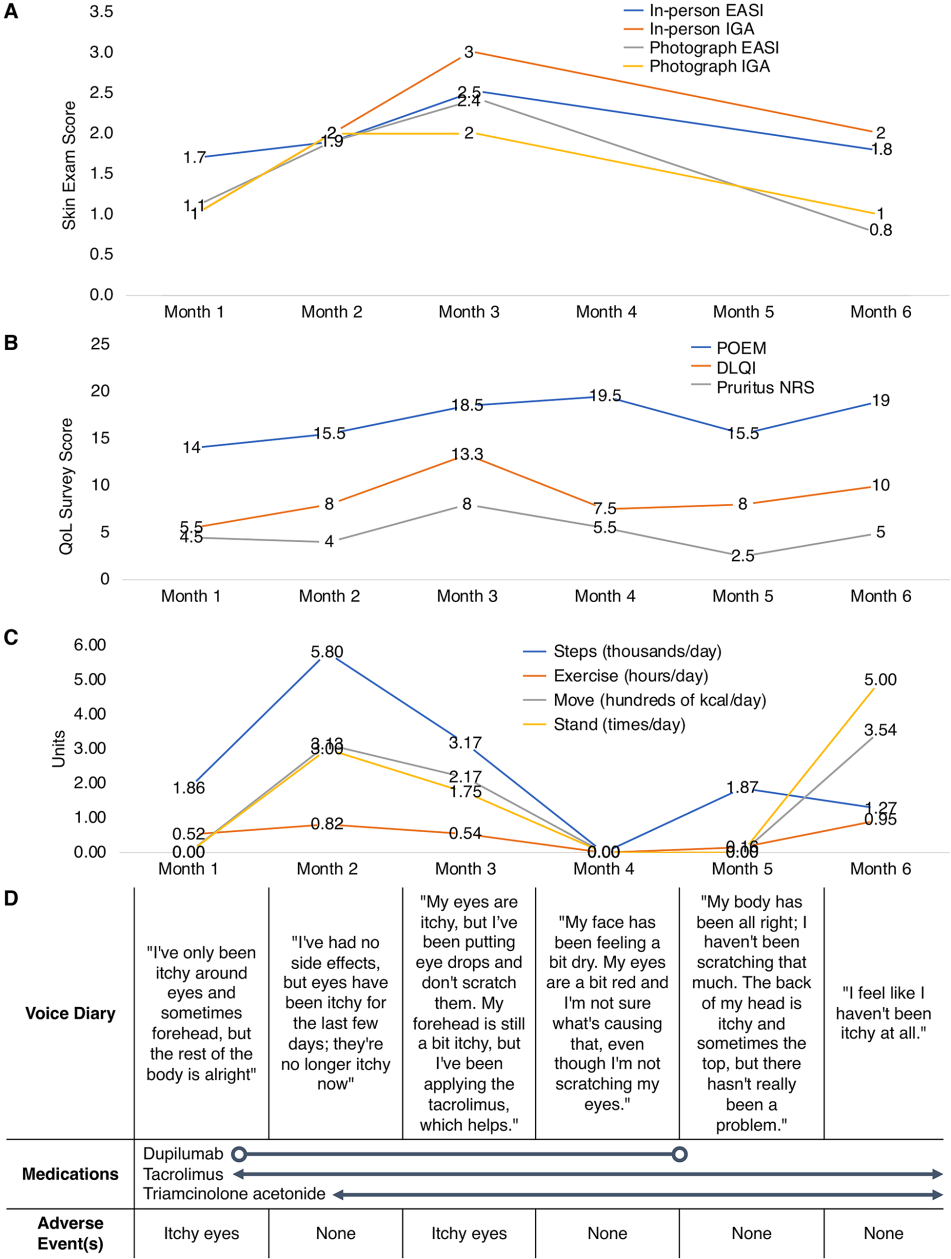
SkinTracker data collected from representative pilot study research subjects. Visualizes the fluctuations in (**A**) clinical outcomes, (**B**) quality-of-life survey data, (**C**) Apple Watch metrics, and (**D**) patient-inputted diary information collected over six months for representative pilot study subjects. The average scores for the POEM, DLQI, and Pruritus NRS are displayed per month when multiple responses (e.g., weekly responses) were available from the subject. DLQI, Dermatology Life Quality Index; EASI, Eczema Area and Severity Index; IGA, Investigator's Global Assessment; NRS, Numerical Rating Scale; POEM, Patient Oriented Eczema Measure.

In-person visits were conducted at Months 0 (eligibility screening, SkinTracker teaching), 1, 2, 3, and 6, while remote subject data was collected continuously throughout the pilot study. At each in-person visit, physical and skin exams were conducted, including EASI and IGA assessments; feedback on the SkinTracker interface, features, and bugs were collected for incorporation in subsequent app updates. Remote EASI/IGA measures, survey data, and Apple Watch information collected through SkinTracker from representative subjects are shown in Figure 5, which displays fluctuations in clinical outcomes and QoL captured over the course of six months.

Patient-submitted photographs were evaluated for image quality along five axes (Table 1), as described by the ImageQX teledermatology quality assessment framework (42). Across 145 submitted photographs, the quality of images was very good in three of five axes, with low frequency of bad framing (3.4%), blur (0%), or problems with distance (2.8%). The two most common image quality issues were bad lighting (34.5%) and low resolution (40.7%). The SkinTracker app user experience was then assessed using a 7-item survey, which included questions evaluating app functionality (learning, navigation, flow logic), aesthetic design, and patient preferences for in-person vs. virtual clinical research participation (Table 2). Nearly all patients agreed or strongly agreed on the fact that using the SkinTracker app made participating in research studies more convenient, compared to all in-person visits; no significant difference in response was noted in pediatric vs. adult subjects. The design and functionality of the app was rated positively.

**Table 1:** Rating of image quality among SkinTracker patient-submitted photographs.

Image quality aspect^a^	Frequency, *n* (%)
Bad framing^b^	5/145 (3.4%)
Blur^c^	0/145 (0.0%)
Distance issue^d^	4/145 (2.8%)
Bad lighting^e^	50/145 (34.5%)
Low resolution^f^	59/145 (40.7%)

^a^ Aspects of poor image quality are extracted from the framework derived by ImageQX, which incorporates input from both dermatologists and a convolutional neural network for image quality assessment in teledermatology (42).

^b^ Bad framing indicates an image not centered on lesions (42).

^c^ Blur indicates an image that suffers from motion blur or inadequate focus (42).

^d^ Distance issue indicates an image where the picture was taken from afar with no details that could be discerned (42).

^e^ Bad lighting indicates an image that is too bright or too dark (42).

^f^ Low resolution indicates an image taken with a low-resolution camera (42).

**Table 2:** SkinTracker pilot study user experience survey results.

Question	User response, mean (SD)^a^
It was easy for me to learn how to use the app	5.50 (2.35)
Overall, the app was easy to use	5.33 (1.75)
I like the interface of the app	5.33 (0.82)
The information in the app was well organized, so I could easily find the information I needed	5.67 (0.82)
It was easy for me to learn how to set up the background and camera equipment needed for skin photography	4.50 (1.97)
Compared to a research study requiring all in-person visits, this app makes participating in research studies more convenient	6.33 (0.82)
I would use this app to participate in future research studies and clinical trials for my skin condition	6.00 (1.26)

SD, standard deviation.

^a^ The mean and SD were calculated after collecting pilot study responses from each subject. The following responses were possible for each question: 1—strongly disagree, 2—disagree, 3—somewhat disagree, 4—neither agree nor disagree, 5—somewhat agree, 6—agree, and 7—strongly agree.

## Discussion

In this study, we describe the development of SkinTracker, a mobile app and web portal system developed via an iterative design process involving dermatologists, clinical research staff, software engineers, graphic designers, and AD patients. Our goal was to develop an integrated digital platform to enable patients to participate in dermatology clinical research remotely. The SkinTracker mobile app and paired wearable smartwatch technology enable skin disease monitoring via photography capture, digital survey completion, and collection of biometric and environmental data. The SkinTracker web portal allows researchers to securely chat with patients, score photographs for skin severity, monitor adverse events and medication use, and collect patient-reported outcomes—data frequently desired in dermatology clinical research (47, 48). Figure 5 illustrates how longitudinal fluctuations in research subjects' skin symptoms and severity can be captured using the mobile app. Patient-submitted photographs were assessed for image quality; bad framing, blur, and distance issues were uncommon—likely resulting from use of standardized anatomic positions, a tripod stand to eliminate camera motion, and a neutral background screen. However, inadequate lighting was found in a significant proportion of images despite user instructions to take photos in an area of good lighting. This may reflect limited lighting options in home environments and suggests that providing subjects with portable external light sources (e.g., ring light) will be important for future studies. Similarly, the presence of low-resolution images was attributed to use of older iPhone models (ranging from the iPhone 8 to 12) with cameras of lower resolution and decreased light sensitivity among some subjects. Future studies could address these limitations by providing research subjects with standardized mobile devices for the duration of the study, or ensuring that personal devices meet minimum camera specifications. At study conclusion, all pilot participants rated their SkinTracker user experience positively on functionality and aesthetic design, and indicated that they would use SkinTracker to participate in future dermatology clinical studies.

The restriction of in-person research activities during the COVID-19 pandemic forced the transition of many interventional studies online to hybrid or fully remote formats (49). Since then, remote clinical studies allowing patients to be home-based at most research stages were associated with higher recruitment rates, improved task compliance, and reduced dropout rates (50). These advantages enabled research investigations to be conducted at a faster pace than traditional studies, for which patient recruitment and dropout issues remain the single biggest cause of delays (3). However, remote clinical trials still represent the vast minority of all clinical trials, in large part due to the lack of solutions allowing for standardized, remote data collection (49).

To our knowledge, few mobile app-based platforms currently exist for the conduct of dermatology clinical research. Review of the literature found reports of four apps developed for AD: one supporting caregivers of children with AD to improve disease self-management (10), an interventional app educating on AD and encouraging adoption of behavioral modifications (11), the myEczema app aiming to collect data on medication prescription/affordability and itch level in eczema patients (13, 14), and a smartwatch app measuring nocturnal scratching in inflammatory skin diseases using accelerometer data (12). The SkinTracker system is distinct in comparison to these published solutions, as it collects a broader range of health behavior data—both active and passive, with validated survey tools and smartwatch biometric activity—and integrates patient-physician interactions (e.g., chat function, physician rating and calculation of EASI and IGA in photographs).

An additional strength of SkinTracker is the relative ease by which it can be customized to study other dermatologic conditions, which would involve use of different disease-specific survey instruments. Thus, SkinTracker may offer improved utility for researchers looking to onboard multiple studies or investigate a wider range of disease outcomes.

While digital apps such as SkinTracker offer a significant opportunity to increase patient recruitment and retention, remote dermatology research still faces several challenges. For example, the epidemiological features of recruited populations in remote research may differ from those of the general population (e.g., elderly participants may be underrepresented due to decreased familiarity with technology). Furthermore, concerns regarding the storage and collection of patient-related health information remain (3); with SkinTracker, our study team prioritized addressing such concerns and worked with our institution's information technology department to review the proposed data storage and data routing process, use institutionally-approved servers, and program secure account creation functions for patients and researchers. Additional challenges observed in remote studies conducted within and outside of dermatology include maintaining research subject motivation and adherence, and ensuring the quality of collected data (3, 49).

In future studies, we plan to continue analyzing dermatologic data collected on the SkinTracker platform in multi-centered studies of patients with AD and other skin conditions with fluctuating disease courses (e.g., psoriasis) to enable robust analyses with increased numbers of patients (15). This pilot study was restricted to use of an iOS mobile app; an Android mobile app equivalent is being developed for inclusion in an upcoming study. Additional modifications are planned for improvement of the SkinTracker app based on pilot subject feedback, including: streamlining the patient photography process with the ultimate goal of reducing the time required to set up equipment and take standardized photos, improving background lighting for higher quality patient photography, collecting additional photos for researcher analysis—especially of evolving lesions on the skin—expanding the range of skin tones photographed (51), and improving the visual design and flow of the SkinTracker app. In upcoming studies, we plan to incorporate infant and child participants, as dermatologic conditions such as AD have a pediatric predilection (1). Finally, data security and privacy are important ethical concerns moving forward. Scoping reviews of mobile health apps have found that the majority share user data with third parties and do not include data sharing information for users (52, 53). To further address such concerns, we plan to incorporate recommendations from a recent study exploring data privacy risks of mobile health apps (54) and provide users with information on data security and app permissions, request explicit permissions when the mobile app is initially opened, improve message confidentiality between patients and research staff, and create an updated Privacy Impact Assessment.

Overall, the development of SkinTracker included patient-centric features discussed between all stakeholders, which was reflected positively in our pilot study user experience survey results. Notably, the mean score of patient responses for all domains were positive, including ease of using the SkinTracker app and learning how to perform the necessary research tasks.

Additional studies are planned utilizing SkinTracker, which will provide an opportunity to further improve and refine the app, with the ultimate goal of benefiting patients and researchers towards the shared goal of improving dermatologic health.

## Data Availability

The original contributions presented in the study are included in the article/**Supplementary Material**, further inquiries can be directed to the corresponding authors.
