# Supplementary material for: Development of SkinTracker, an integrated dermatology mobile app and web portal enabling remote clinical research studies
Source: Front Digit Health. 2023 Sep 6;5:1228503. doi: 10.3389/fdgth.2023.1228503 (PMC10516539; doi:10.3389/fdgth.2023.1228503)

**SUPPLEMENTARY MATERIAL**

**Table of Contents**

**Supplemental Methods.** User surveys included in the Skintracker app.

**Supplementary Figure 1.** SkinTracker data diagram for security.

**Supplemental Methods. User surveys included in the Skintracker app.**

1. Dermatology life quality survey (DLQI):

- Over the last week, how itchy, sore, painful or stinging has your skin been? [Select one: very much, a lot, a little, not at all, not relevant]
- Over the last week, how embarrassed or self conscious have you been because of your skin? [Select one: very much, a lot, a little, not at all, not relevant]
- Over the last week, how much has your skin interfered with you going shopping or looking after your home or yard? [Select one: very much, a lot, a little, not at all, not relevant]
- Over the last week, how much has your skin influenced the clothes you wear? [Select one: very much, a lot, a little, not at all, not relevant]
- Over the last week, how much has your skin affected any social or leisure activities? [Select one: very much, a lot, a little, not at all, not relevant]
- Over the last week, how much has your skin made it difficult for you to do any sport? [Select one: very much, a lot, a little, not at all, not relevant]
- Over the last week, has your skin prevented you from working or studying? [Select one: yes, no, not relevant]
- Over the last week, how much has your skin been a problem at work or studying? [Select one: a lot, a little, not at all]
- Over the last week, how much has your skin created problems with your partner or any of your close friends or relatives? [Select one: very much, a lot, a little, not at all, not relevant]
- Over the last week, how much has your skin caused any sexual difficulties? [Select one: very much, a lot, a little, not at all, not relevant]
- Over the last week, how much of a problem has the treatment for your skin been, for example by making your home messy, or by taking up time? [Select one: very much, a lot, a little, not at all, not relevant]

2. Pruritus (i.e., itch) numerical rating scale (NRS) survey:

- On a scale from 0 (no itch) to 10 (worst imaginable itch), how was your itch, on average, in the past 24 hours? Please select one number. [Select a number between 0-10]
- On a scale from 0 (no itch) to 10 (worst imaginable itch), how was your worst itch in the past 24 hours? Please select one number. [Select a number between 0-10]

3. Patient Oriented Eczema Measure (POEM), i.e., eczema severity survey:

- Over the last week, on how many days has your skin been itchy because of your eczema? [select one: no days, 1-2 days, 3-4 days, 5-6 days, every day]
- Over the last week, on how many nights has your sleep been disturbed because of your eczema? [select one: no days, 1-2 days, 3-4 days, 5-6 days, every day]
- Over the last week, on how many days has your skin been bleeding because of your eczema? [select one: no days, 1-2 days, 3-4 days, 5-6 days, every day]
- Over the last week, on how many days has your skin been weeping or oozing clear fluid because of your eczema? [select one: no days, 1-2 days, 3-4 days, 5-6 days, every day]
- Over the last week, on how many days has your skin been cracked because of your eczema? [select one: no days, 1-2 days, 3-4 days, 5-6 days, every day]
- Over the last week, on how many days has your skin been flaking off because of your eczema? [select one: no days, 1-2 days, 3-4 days, 5-6 days, every day]
- Over the last week, on how many days has your skin felt dry or rough because of your eczema? [select one: no days, 1-2 days, 3-4 days, 5-6 days, every day]

4. Medication usage survey:

- Please enter a prescription medication you are taking. Over-the-counter (OTC) medications and supplements can be entered in the next category for questions. [Free text]
- When did you start using this medication? [Select date: month, day, year]
- Why do you use this medication? [Free text]
- How do you take your medication? [Select any number: liquid, tablet, capsule, topical, suppository, inhaler, injection, implant or patch, intravenous]
- How much of this medication are you taking? [Select one: X dose (enter number and concentration), don’t know, topically applied]
- How often do you take your medication? [Select for X and Y: X times every Y period]
- How did your skin respond? [Select one: much better, slightly better, no effect, worse]
- Would you like to list another medication you are currently using? [Select: yes, no]
  - If yes, repeat questions above
  - If no:
    - Would you like to list supplements that you are currently using? [Select one: yes, no]
      - If yes:
        - Please enter the name of the supplement you are taking. [Free text]
        - When did you start using this medication? [Select date: month, day, year]
        - Why do you use this medication? [Free text]
        - How do you take your medication? [Select any number: liquid, tablet, capsule, topical, suppository, inhaler, injection, implant or patch, intravenous]
        - How often do you take your medication? [Select for X and Y: X times every Y period]
        - Would you like to list another over-the-counter (OTC) medication or supplement that you are currently using? [Select one: yes, no]

If yes, repeat questions for supplements above

5. Adverse events survey

- Do you have any side effects? [Select one: yes, no]
  - If yes:
    - Describe side effect [Free text]
    - Date of onset of side effect [Select date: month, day, year]
    - Frequency of the side effect [Select one: daily, once a week, a few times a week, monthly]
    - Resolved? [Select one: yes, ongoing]
    - Date of resolution? [Select date: month, day, year]
    - Have you seen a medical provider for this problem? [Select one: yes, no]
    - Name of provider [Free text]
    - Specialty of provider [Free text]
    - Hospital or institution [Free text]
    - Are you currently treating this problem? [Select one: yes, no]
      - If yes:
        - Which treatment are you using? [Free text]
        - Route [Select one: liquid, tablet, capsule, topical, suppository, inhaler, injection, implant, patch, intravenous]
        - Dose [Enter number/concentration: X dose]
        - Frequency [Select for X and Y: X times every Y period]
        - Would you like to submit photographs for the study team to review? [Select one: yes, no]

If yes, upload photo(s)

**Supplementary Figure 1.** SkinTracker data diagram for security.


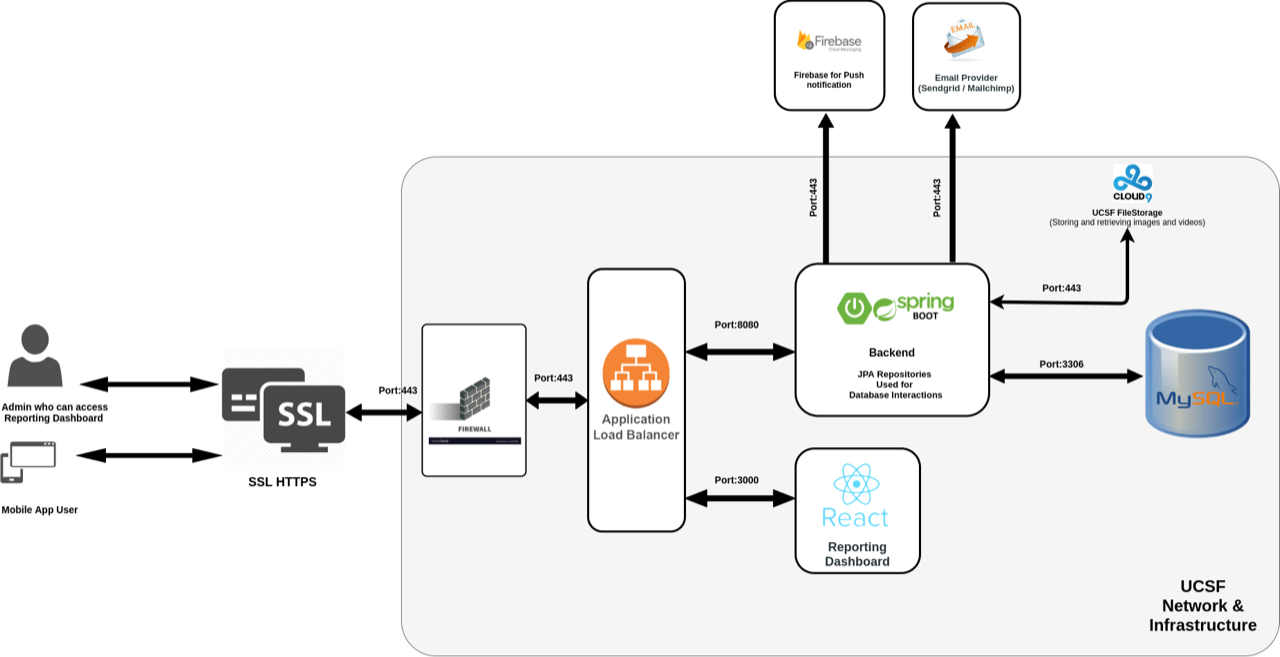

Supplement: Supplementary file 1 [file Datasheet1.docx]
